# Smaller Hippocampal Volume and Degraded Peripheral Hearing Among Japanese Community Dwellers

**DOI:** 10.3389/fnagi.2018.00319

**Published:** 2018-10-16

**Authors:** Yasue Uchida, Yukiko Nishita, Takashi Kato, Kaori Iwata, Saiko Sugiura, Hirokazu Suzuki, Michihiko Sone, Chikako Tange, Rei Otsuka, Fujiko Ando, Hiroshi Shimokata, Akinori Nakamura

**Affiliations:** ^1^Department of Otolaryngology, Aichi Medical University, Nagakute, Japan; ^2^Department of Otorhinolaryngology, National Center for Geriatrics and Gerontology, Obu, Japan; ^3^Section of NILS-LSA, National Center for Geriatrics and Gerontology, Obu, Japan; ^4^Department of Clinical and Experimental Neuroimaging, National Center for Geriatrics and Gerontology, Obu, Japan; ^5^Department of Otorhinolaryngology, Nagoya University Graduate School of Medicine, Nagoya, Japan; ^6^Department of Health and Medical Sciences, Aichi Shukutoku University, Nagakute, Japan; ^7^Graduate School of Nutritional Sciences, Nagoya University of Arts and Sciences, Nisshin, Japan

**Keywords:** hearing, Freesurfer, hippocampus, Heschl’s gyrus, gray matter

## Abstract

A growing body of literature has demonstrated that dementia and hearing loss are interrelated. Recent interest in dementia research has expanded to brain imaging analyses with auditory function. The aim of this study was to investigate the link between hearing ability, which was assessed using pure-tone audiometry, and the volume of brain regions, specifically the hippocampus, entorhinal cortex, Heschl’s gyrus, and total gray matter, using Freesurfer software and T1-weighted brain magnetic resonance imaging. The data for 2082 samples (age range = 40–89 years) were extracted from a population-based cohort of community dwellers. Hearing-impaired individuals showed significantly smaller hippocampal volumes compared with their non-hearing-impaired counterparts for all auditory frequency ranges. In addition, a correlational analysis showed a significant dose-response relationship for hearing ability and hippocampal volume after adjusting for potential confounding factors so that the more degraded the peripheral hearing was, the smaller the hippocampal volume was. This association was consistent through the auditory frequency range. The volume of the entorhinal cortex, right Heschl’s gyrus and total gray matter did not correlate with hearing level at any frequency. The volume of the left Heschl’s gyrus showed a significant relationship with the hearing levels for some auditory frequencies. The current results suggested that the presence of hearing loss after middle age could be a modifier of hippocampal atrophy.

## Introduction

Recently, the number of studies investigating the interrelationship of dementia and hearing loss has increased rapidly. Systematic reviews and meta-analyses of the association between hearing loss and cognitive function, cognitive impairment, and dementia were successively published in 2017 (Loughrey et al., 2017; Thomson et al., 2017; Zheng et al., 2017). Loughrey et al. (2017) found a significant association between age-related hearing loss and 10 cognitive domains, such as global cognition, executive function, and episodic memory, in 26 cross-sectional studies involving 15,620 participants and 1185 records. Similar results have been reported by nine cohort studies. Although other systematic reviews had different inclusion criteria, analytical estimates have suggested that hearing loss might be a biomarker and modifiable risk factor for cognitive impairments and dementia in older adults.

A novel lifespan-based model of dementia risk was presented by the Lancet Commission on Dementia Prevention, Intervention, and Care at the 2017 Alzheimer’s Association International Conference in London and simultaneously published in *Lancet* (Livingston et al., 2017). The Commission estimated a combined PAF for known modifiable risk factors for dementia and reported that nine potentially modifiable life course risk factors might prevent about 35% of global dementia cases. Of the nine risk factors, hearing loss had the highest PAF value, and hearing loss management results in a 9% reduction in the incidence of dementia. The PAF, which is a statistical concept that can be used to quantify the impact of exposure to a certain risk factor at the population level, estimates the percentage of disease cases in a given population that would theoretically not have occurred if none of the individuals had been exposed to the risk factor. Under strict criteria during the analytical process, the Commission conducted a new review and meta-analysis, in which a cohort of cognitively healthy people was followed for at least 5 years, an objective measure of peripheral hearing (pure-tone audiometry) was obtained, incident dementia was calculated as an outcome, and potential confounding factors were adjusted. The pooled relative risk of hearing loss for dementia in the meta-analysis of three studies with follow-ups over 9, 12, and 17 years was 1.94 (95% confidence interval: 1.38–2.73).

Despite the numerous epidemiological studies of this association, the causal link between hearing loss and the increased risk of developing dementia has not yet been clarified. Recent research studies have focused on the imaging analysis of the aged brain relative to auditory function. Several morphological studies have been conducted to understand the relevance of hearing loss to brain volume. The cerebral regions that have been studied regarding hearing loss have varied enormously, and the results have not been consistent. The central auditory pathway is one of the most commonly studied functional structures. A relationship between hearing loss and decreased GM volume in the primary auditory cortex region has been found (Peelle et al., 2011; Eckert et al., 2012), while no relationship between hearing loss and brain volume has also been reported (Profant et al., 2014). One large population-based cross-sectional study has reported an association between hearing loss and decreased total brain volume, especially WM volume (Rigters et al., 2017). Another study of longitudinal setting has demonstrated that individuals with baseline hearing loss had accelerated rates of atrophy in the whole brain and temporal lobe GM compared to individuals with normal hearing (Lin et al., 2014).

In the current study, we specifically investigated the link between hippocampal volume and hearing ability for the following reasons. First, the hippocampus is a key structure of memory processing, the disturbance of which is one of the core symptoms of dementia. In addition, previous cross-sectional studies have reported decreased memory performance in hearing-impaired individuals compared with individuals who were not hearing impaired (Deal et al., 2017) and faster rates of cognitive memory decline in people with hearing loss compared to those without hearing loss (Valentijn et al., 2005; Deal et al., 2015). Second, hippocampal atrophy occurs during the disease progression of Alzheimer’s disease and several non-Alzheimer’s dementing disorders, including dementia with grains, hippocampal sclerosis (Malmgren and Thom, 2012), and senile dementia of the neurofibrillary tangle type (Jellinger and Attems, 2007). Third, several animal studies have shown that hearing loss is intimately involved in hippocampal neuropathological alteration and dysfunction. For example, age-related hearing loss is accompanied by the degeneration of synapses in the hippocampal CA3 region of C57BL/6J mice (Yu et al., 2011), and noise-induced hearing loss is accompanied by memory dysfunction and decreased hippocampal neurogenesis in mice and rats (Kraus et al., 2010; Liu et al., 2016; Park et al., 2018).

Little is known about the relationship between hippocampal volume and hearing ability in humans. Therefore, in this study, we examined the detailed relationships between hearing ability, which was assessed by pure-tone audiometry, and hippocampal volume, which was estimated by T1-weighted MRI using Freesurfer software in 2082 community-dwelling volunteers who were 40–89 years old. In addition, we analyzed volumes of the entorhinal cortex, which is also an important structure for the memory function, and known to be a major hub within the medial temporal lobe that mediates hippocampal neocortical communication. Further, we analyzed the relationships between hearing ability and total GM volume and the volume of Heschl’s gyrus as references.

## Materials and Methods

### Study Subjects and Protocol

The participants analyzed in the present study were from the NILS-LSA, a population-based biennial survey of a cohort of approximately 2300 adults. The NILS-LSA is a comprehensive and interdisciplinary study that evaluates age-related changes using detailed questionnaires; medical examinations; blood chemical analyses; and body composition, anthropometry, physical function, nutritional analysis, psychological, and visual and auditory function tests.

The NILS-LSA participants were community-dwellers in Aichi Prefecture in central Japan who were randomly selected from resident registrations and stratified by both age and sex in cooperation with the local government. The numbers of males and females recruited were similar, and their ages at the first-wave examination (November 1997–April 2000) ranged from 40 to 79 years, with similar numbers of participants in each decade of age (40s, 50s, 60s, and 70s). The cohort design was dynamic, and new participants with ages in their 40s were recruited every year.

The data analyzed in the present study were collected from the participants (age range = 40–89 years) at the sixth-wave examination (July 2008–July 2010) of the NILS-LSA because the three-dimensional MRI data (explained below) of the NILS-LSA were available after the sixth wave.

The study protocol was approved by the Committee of Ethics of Human Research of the National Center for Geriatrics and Gerontology (approval numbers 899-2). Written informed consent was obtained from all participants. The details of the NILS-LSA have been described elsewhere (Shimokata et al., 2000).

### Brain MRI Acquisition and Cortical Volume Measurements

All MRI scans were performed on a MAGNETOM Trio 3T system (Siemens Healthcare GmbH, Erlangen, Germany). Three-dimensional T1 MRI scans were acquired using a volumetric T1-weighted magnetization-prepared rapid gradient-echo sequence (Repetition time/Echo time/Inversion time = 1800/1.98/800 ms, 9° flip angle, 0.98 × 0.98 × 1.1 mm^3^ resolution, and 256 × 256 matrix).

FreeSurfer (version 5.3)^1^ was used for the cortical surface reconstructions and regional GM volume estimations. The technical details of these procedures have been described previously (Fischl et al., 1999; Fischl and Dale, 2000; Fischl et al., 2004). In short, the fully automated procedure in FreeSurfer involves preprocessing the subject’s image data, segmenting the cortical GM and WM (GM/WM), tessellating the GM/WM junction, inflating the folded surface tessellation patterns, and automatically correcting the topological defects. Subsequently, FreeSurfer parcellated the cerebral cortex into gyral-based regions of interest according to the Desikan-Killiany atlas (Desikan et al., 2006; Destrieux et al., 2010) and performed an automatic subcortical segmentation (Fischl et al., 2002). If any of the FreeSurfer processes, such as segmentation, failed, the data for those cases were excluded from the analyses. The volumes (mm^3^) of the left-right summation of the hippocampus, the left-right summation of the entorhinal cortex, right and left transverse temporal gyri (Heschl’s gyri), and total GM were the dependent measures. To account for individual differences in head size and express in the unit of mm^3^, individual regional GM volume was normalized by dividing it by the estimated total intracranial volume and then multiplying by the mean estimated total intracranial volume of all subjects.

### The Audiometric Test and Other Measures

Air conduction pure-tone thresholds were measured at octave intervals from 0.125 to 8 kHz in a sound-proof booth by trained examiners using a standardized protocol and a diagnostic audiometer (AA-78; RION Co., Ltd., Tokyo, Japan). In the analysis, we used the hearing thresholds of the ear with the better hearing that was based on the PTA thresholds of the following frequency ranges: low frequency (average of 0.125 and 0.25 kHz: PTA low), speech range (average of 0.50, 1, 2, and 4 kHz: PTA speech), and high frequency (average of 4, 6, and 8 kHz: PTA high). Hearing impairment was defined in the analysis as PTA low, PTA speech, and PTA high scores over 25 dB, which indicates bilateral hearing loss.

The participants completed a series of questionnaires before the examination. Years of education (years of school); smoking status (yes or no); and medical history of hypertension, hyperlipidemia, stroke, cardiac disease, and/or diabetes (yes or no) were recorded. Alcohol consumption in the previous year was assessed using a food frequency questionnaire (conversion into ethanol, mL/day). For the level of education in Japan, nine or less indicates elementary school or junior high school equivalent, 10–12 indicates high school or junior high school equivalent under the former Japanese educational system, 13–14 is comparable to higher vocational school or junior college, and 15 or more years is comparable to college or graduate college. Depressive symptoms were assessed by the Center for Epidemiologic Studies Depression Scale, and the cutoff value was set to 15/16 points.

### Statistical Analyses

The statistical analyses were conducted using Statistical Analysis System (SAS) software (version 9.3; SAS Institute, Inc., Cary, NC, United States).

For the univariate analysis, a *t*-test was used to compare differences in the continuous variables between the two groups, and comparisons of categorical variables were done using the chi-square test. Unless otherwise noted, the values are expressed as mean ± standard deviation (SD). Comparison of the regional brain volumes based on the presence or absence of hearing impairment in the PTA low, PTA speech, and PTA high frequency ranges was made using Tukey-Kramer multiple comparison tests. During the multivariate approach, age; sex; smoking status; alcohol consumption; years of education; medical history of hypertension, hyperlipidemia, cardiac disease, and diabetes; and depressive symptoms were consistently used as adjustment variables.

Scatterplots were employed to illustrate an overview of the potential association between each region of brain volume and PTA speech. Single and multiple regressions (adjustments were made for the 10 aforementioned variables) were analyzed using the General Linear Model (GLM) procedure of the SAS software. In addition, the PROC REG procedure in SAS was performed to obtain standardized partial regression coefficients in the relationship between each PTA and regional brain volume. Each region of brain volume was set as the dependent variable with adjustments for the 10 aforementioned variables. The standardized partial regression coefficients of brain volume were calculated per decibel increase in the hearing thresholds for PTA low, PTA speech, and PTA high frequency ranges. *P* values less than 0.05 were considered significant.

## Results

**FIGURE 1:**
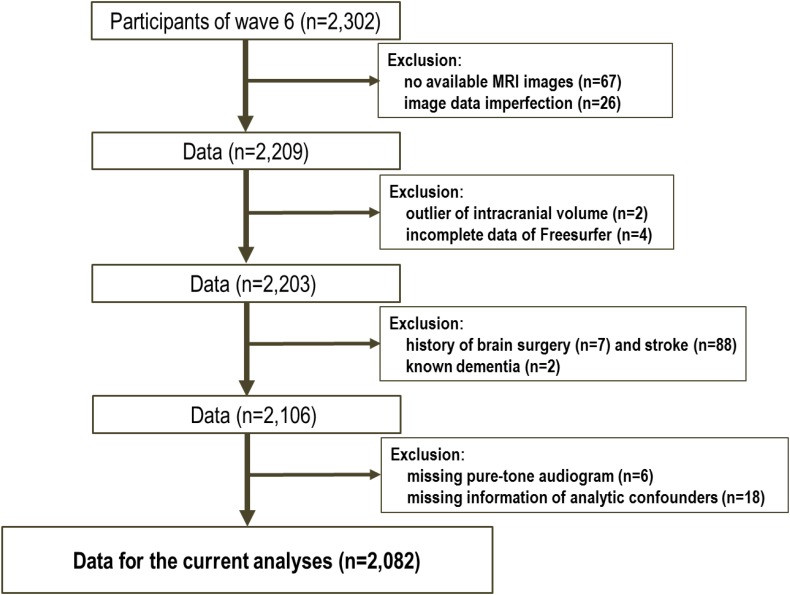
Flow chart of subject selection.

The data for the 2302 participants were extracted from the sixth-wave examination of the NILS-LSA. Following the analysis preparation process, 220 subjects were excluded (see **Figure 1**); therefore, 2082 samples were included in this study. **Table 1** shows the demographic characteristics of the study population. The mean age of the subjects was 61.0 years (SD: 12.4), and 49.8% were male. According to the univariate analysis of the demographic profiles according to the category of hearing impairment for PTA low, PTA speech, and PTA high frequency ranges, significant differences between those with and without hearing impairments were observed in mean age, educational level, and comorbidity (i.e., hypertension, diabetes, and so on). For the percentage of male participants and smokers, the significance differed in each classification.

**Table 1:** Demographic characteristics of the study population (*N* = 2082).

	*N* = 2082	PTA low (0.125, 0.25 kHz)			PTA speech (0.5, 1, 2, 4 kHz)			PTA high (4, 6, 8 kHz)		
			
Characteristic		No hearing impairment *N* = 1563	Hearing impairment *N* = 519	*p*-value	No hearing impairment *N* = 1544	Hearing impairment *N* = 538	*p*-value	No hearing impairment *N* = 1204	Hearing impairment *N* = 878	*p*-value
Mean age, year	61.0 ± 12.4	57.8 ± 11.2	70.7 ± 10.5	<0.0001	56.9 ± 10.7	72.7 ± 8.9	<0.0001	54.4 ± 9.6	70.1 ± 9.7	<0.0001
PTA speech, dB	19.9 ± 12.5	15.9 ± 8.8	31.9 ± 14.1	<0.0001	13.8 ± 5.6	37.3 ± 10.3	<0.0001	12.3 ± 5.2	30.3 ± 12.0	<0.0001
Education, year	12.7 ± 2.8	13.1 ± 2.6	11.5 ± 2.8	<0.0001	13.2 ± 2.5	11.2 ± 2.8	<0.0001	13.5 ± 2.5	11.5 ± 2.7	<0.0001
Alcohol consumption, ml/day	13.8 ± 24.3	13.9 ± 23.8	13.8 ± 25.9	0.9426	14.2 ± 24.5	12.9 ± 23.8	0.2791	13.0 ± 22.9	15.0 ± 26.1	0.0689
Sex, male [n (%)]	1036 (49.8)	767 (49.1)	269 (51.8)	0.2762	735 (47.6)	301 (56.0)	0.0009	523 (43.4)	513 (58.4)	<0.0001
Hypertension [n (%)]	574 (27.6)	348 (22.3)	226 (43.6)	<0.0001	332 (21.5)	242 (45.0)	<0.0001	226 (18.8)	348 (39.6)	<0.0001
Hyperlipidemia [n (%)]	417 (20.0)	293 (18.8)	124 (23.9)	0.0111	290 (18.8)	127 (23.6)	0.0161	217 (18.0)	200 (22.8)	0.0074
Diabetes [n (%)]	152 (7.3)	96 (6.1)	56 (10.8)	0.0004	87 (5.6)	65 (12.1)	<0.0001	55 (4.6)	97 (11.1)	<0.0001
Cardiac disease [n (%)]	74 (3.6)	37 (2.4)	37 (7.1)	<0.0001	36 (2.3)	38 (7.1)	<0.0001	16 (1.3)	58 (6.6)	<0.0001
Smoker [n (%)]	292 (14.0)	237 (15.2)	55 (10.6)	0.0094	227 (14.7)	65 (12.1)	0.1317	168 (14.0)	124 (14.1)	0.9124
Depressive symptoms [n (%)]	263 (12.6)	196 (12.5)	67 (12.9)	0.8262	184 (11.9)	79 (14.7)	0.0962	145 (12.0)	118 (13.4)	0.3435


*Abbreviations: PTA, pure-tone average. PTA low (0.125, 0.25 kHz): the average hearing thresholds of the low frequencies (0.125, 0.25 kHz) in the better hearing ear. PTA speech (0.5, 1, 2, 4 kHz): the average hearing thresholds of the speech frequencies (0.5, 1, 2, 4 kHz) in the better hearing ear. PTA high (4, 6, 8 kHz): the average hearing thresholds of the speech frequencies (4, 6, 8 kHz) in the better hearing ear. Hearing impairment was defined as a PTA greater than 25 dB. Unless stated, the values are presented as mean ± standard deviation (SD) for continuous variables or numbers (percentage) for dichotomous variables.*

The results of the comparison of regional brain volumes according to the presence or absence of hearing impairments at the PTA low, PTA speech, and PTA high frequency ranges are shown in **Table 2**. Significant differences were found in hippocampal volume between those with and without hearing impairment across the auditory frequency range. Hearing-impaired individuals showed significantly smaller hippocampal volumes compared with the non-hearing-impaired counterparts after adjusting for potential confounding factors. The volume of the left Heschl’s gyrus differed significantly according to hearing status for only the PTA high range. No significant difference was observed in the other regional brain volumes.

**Table 2:** Comparison of the presence or absence of hearing impairment and regional brain volumes using the Tukey–Kramer multiple comparison test.

	PTA low (0.125, 0.25 kHz)			PTA speech (0.5, 1, 2, 4 kHz)			PTA high (4, 6, 8 kHz)		
			
Least squares mean of regional brain volumes (mm^3^)	No hearing impairment	Hearing impairment	*p*-value	No hearing impairment	Hearing impairment	*p*-value	No hearing impairment	Hearing impairment	*p*-value
Hippocampus	**8698.6**	**8589.9**	**0.030**	**8712.1**	**8563.4**	**0.006**	**8725.5**	**8606.4**	**0.021**
Entorhinal cortex	2232.5	2237.5	0.804	2243.1	2211.1	0.138	2242.7	2224.0	0.370
Right Heschl’s gyrus	698.4	703.0	0.552	701.6	694.9	0.417	705.6	693.0	0.117
Left Heschl’s gyrus	917.8	917.0	0.931	923.1	903.9	0.059	**934.0**	**899.2**	**<0.001**
Gray matter	541335.8	540758.5	0.738	541709.9	539867.8	0.318	542797.6	539365.0	0.054


*Abbreviations: PTA, pure-tone average. PTA low (0.125, 0.25 kHz): the average hearing thresholds of the low frequencies (0.125, 0.25 kHz) in the better hearing ear. PTA speech (0.5, 1, 2, 4 kHz): the average hearing thresholds of the speech frequencies (0.5, 1, 2, 4 kHz) in the better hearing ear. PTA high (4, 6, 8 kHz): the average hearing thresholds of the speech frequencies (4, 6, 8 kHz) in the better hearing ear. Hearing impairment was defined as a PTA greater than 25 dB. Adjusted for age; sex; smoking status; alcohol consumption; years of education; medical history of hypertension, hyperlipidemia, cardiac disease, and diabetes; and depressive symptoms. Bold values indicate statistically significant results.*

**FIGURE 2:**
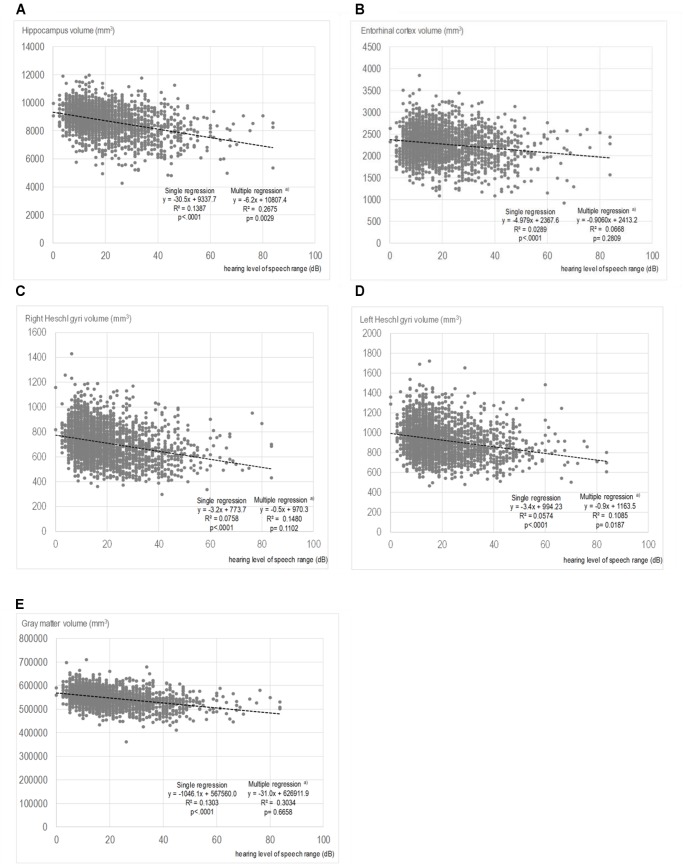
Scatter plots showing the relationships between the volumes of each region and pure-tone average speech. **(A)** Hippocampus volume and PTA speech. **(B)** Entorhinal cortex volume and PTA speech. **(C)** Right Heschl’s gyrus volume and PTA speech. **(D)** Left Heschl’s gyrus volume and PTA speech. **(E)** Gray matter volume and PTA speech. In the multiple regressions, adjustments were made for age; sex; smoking status; alcohol consumption; years of education; medical history of hypertension, hyperlipidemia, cardiac disease, and diabetes; and depressive symptoms

The results of the correlational analysis are shown on scatterplot diagrams to illustrate the relationships between each region of brain volume and the PTA. Only the diagrams for PTA speech are shown as a representative example. **Figures 2A–E**) reveal the relationships and results of the single and multiple regression analyses of the PTA speech range with the volumes of the hippocampus, the entorhinal cortex, right Heschl’s gyrus, left Heschl’s gyrus, and total GM. The correlational analysis with the single regression model showed that the PTA speech range inversely correlated with the hippocampal volume (regression coefficient: -30.5, *p* < 0.0001, **Figure 2A**). Similarly, in the other regions, greater hearing impairments were associated with smaller brain volumes. In the multiple regression model, significance was still observed for the volumes of the hippocampus and left Heschl’s gyrus, even after adjusting for possible confounders.

The results of the multiple regression analysis of the associations between the hearing level of each frequency range and the volumes of multiple brain regions are presented in **Table 3**. Hippocampal volume had a significant relationship with hearing level, regardless of the frequency range. The standardized partial regression coefficients were -0.0509 for the PTA low range (*p* = 0.0193), -0.0758 for the PTA speech range (*p* = 0.0029), and -0.0917 for the PTA high range (*p* = 0.0011). The volumes of the entorhinal cortex, right Heschl’s gyrus and total GM did not show significant relationships with the hearing levels for all frequency ranges. The volume of the left Heschl’s gyrus showed a significant relationship with the hearing levels for the PTA speech and PTA high ranges. The values of the goodness-of-fits of the analytical model were highest for total GM volume (0.3034 for PTA low, 0.3034 for PTA speech, and 0.3040 for PTA high) and second highest for hippocampal volume (0.2663 for PTA low, 0.2675 for PTA speech, and 0.2681 for PTA high).

**Table 3:** The results of the multiple regression analyses of the relationships between hearing level and brain volume.

Independent variables									

	PTA low (0.125, 0.25 kHz)			PTA speech (0.5, 1, 2, 4 kHz)			PTA high (4, 6, 8 kHz)		
			
Dependent variable	Standardised partial regression coefficient	*p*	Goodness of fit in the model R^2^	Standardised partial regression coefficient	*p*	Goodness of fit in the model R^2^	Standardised partial regression coefficient	*p*	Goodness of fit in the model R^2^
Hippocampus	**-0.0509**	**0.0193**	0.2663	**-0.0758**	**0.0029**	0.2675	**-0.0917**	**0.0011**	0.2681
Entorhinal cortex	0.0243	0.3215	0.0667	-0.0309	0.2809	0.0668	-0.0362	0.2531	0.0669
Right Heschl’s gyrus	-0.0138	0.5572	0.1471	-0.0438	0.1102	0.1480	-0.0489	0.1066	0.1480
Left Heschl’s gyrus	-0.0295	0.2190	0.1068	**-0.0660**	**0.0187**	0.1085	**-0.1104**	**0.0004**	0.1116
Gray matter	-0.0109	0.6083	0.3034	-0.0107	0.6658	0.3034	-0.0397	0.1469	0.3040


*Abbreviations: PTA, pure-tone average. PTA low (0.125, 0.25 kHz): the average hearing thresholds of the low frequencies (0.125, 0.25 kHz) in the better hearing ear. PTA speech (0.5, 1, 2, 4 kHz): the average hearing thresholds of speech frequencies (0.5, 1, 2, 4 kHz) in the better hearing ear. PTA high (4, 6, 8 kHz): the average hearing thresholds of the speech frequencies (4, 6, 8 kHz) in the better hearing ear. Outcome indicates the standardized partial regression coefficient of the brain volume of each region per decibel increase in hearing threshold. Adjusted for age; sex; smoking status; alcohol consumption; years of education; medical history of hypertension, hyperlipidemia, cardiac disease, and diabetes; and depressive symptoms. Bold values indicate statistically significant results.*

## Discussion

The present epidemiological study of over 2000 community dwellers aged 40 or older demonstrated that peripheral hearing ability assessed by pure-tone audiometry significantly correlated with hippocampal volume after adjusting for potential confounding factors. Hearing-impaired individuals showed significantly smaller hippocampal volumes compared with their non-hearing-impaired counterparts for all auditory frequency ranges. In addition, a correlational analysis showed a significant dose-response relationship for hearing ability and hippocampal volume. Throughout the auditory frequency range, the worse the hearing, the smaller the hippocampal volume. Total GM volume did not significantly correlate with hearing ability at any frequency range.

Although the correlational analysis in the single regression model showed a significant inverse association of hearing threshold with the brain volume of each targeted region, the data might have resulted from confounding effects. Aging, in particular, influences the volumes and structures of the brain (Fjell and Walhovd, 2010; Tabatabaei-Jafari et al., 2015) and strongly affects hearing ability (Gates and Mills, 2005). In fact, the effects of hearing on the volumes of the entorhinal cortex, right Heschl’s gyrus and total GM disappeared after adjusting for the effects of confounders, including age. Notably, the significant link between hearing degradation and small hippocampal volume was found even after the adjustments in the multivariate approach.

Few studies have examined the relationship between age-related hearing loss and brain morphology using MRI. The results of previous studies were not always consistent with our result. Peelle et al. (2011) have examined the relationship between hearing ability, which was measured using the pure tone average threshold in each participant’s better ear for 1, 2, and 4 kHz, to cortical structural integrity using voxel-based morphometry in 25 adults between the ages of 60 and 77 (Peelle et al., 2011). They demonstrated a significant linear relationship between hearing ability and GM volume for the right primary auditory cortex and a similar nonsignificant trend in left auditory cortex. An additional exploratory whole-brain analysis did not find any regions outside the auditory cortex that were significantly related to hearing ability.

Rigters et al. (2017) have examined the relationship between hearing impairments and brain volume in the population-based Rotterdam Study, which included 2908 participants (mean age: 65 years; 56% female). They quantified hearing impairments for the best hearing ear by taking the average threshold over all frequencies, namely 0.25, 0.50, 1, 2, 4, and 8 kHz (Rigters et al., 2017). They found that hearing impairments were associated with small total brain volume, which was driven by small WM volumes and which was consistent across the hearing frequencies.

In the Baltimore Longitudinal Study of Aging, Lin et al. (2014) followed 126 adults (age, 56–86 years) for a mean of 6.4 years after baseline MRI scans, and they found that individuals with audiometric hearing loss had faster declines in brain volume over time compared with that of normal hearing individuals. Significantly accelerated volume decreases were noted in the whole brain and regions in the right temporal lobe in individuals with hearing loss compared with normal hearing individuals. They defined audiometric hearing loss as the speech-frequency PTAs of the air conduction thresholds at 0.5, 1, 2, and 4 kHz that were greater than 25 dB in the better-hearing ear. They compared the baseline global and lobar brain volumes and their annual rates of change in brain regions, including the hippocampus, parahippocampus, and entorhinal cortex, between individuals with and without hearing impairments after adjusting for possible confounders. Compared to individuals with normal hearing, individuals with hearing impairments exhibited accelerated volume declines in the superior, middle, and inferior temporal gyri and parahippocampal gyrus of the right, but not the left, temporal lobe.

The present results of the multiple regression analysis showed that the volume of the left Heschl’s gyrus, but not the right Heschl’s gyrus, was significantly related to the hearing levels for the PTA speech and PTA high frequencies. Lin et al. (2014) have interpreted their results as indicating that the more frequent processing of auditory signals in the left temporal lobe, which occurs because spoken language is predominantly processed in the left temporal lobe (Peelle, 2012), might preserve the structural volume of the left temporal lobe. Peelle et al. (2011) have also suggested a link between sensory stimulation and cortical volume from their finding that GM density in primary auditory areas is predicted by peripheral hearing ability. Our results showing a significant relationship between hearing ability with the volume of the left Heschl’s gyrus volume do not contradict their speculation, but further analyses by longitudinal design will be required in order to show whether the cause precedes the effect.

Several hypotheses of how hearing loss is related to cognitive decline have been proposed (Wayne and Johnsrude, 2015; Stahl, 2017; Uchida et al., 2018). Multiple hypotheses are based on the common cause or shared neurobiological pathology hypothesis, such as the cognitive load hypothesis, which suggests that brain resources are reallocated for effortful listening and auditory processing. The cascade or psychosocial hypothesis proposes that impoverished auditory inputs and poor verbal communication cascades into decreased socialization and depression, but the direction of a potential causal arrow between depression and the hippocampus still remains unresolved (Sheline, 2011).

One of these hypothetical mechanisms could explain the mutual relationship between degraded hearing and hippocampal atrophy. A common neuropathologic process that causes declines in both hearing and cognition might affect both cochlear and brain aging. Those potential common factors include oxidative stress, microcirculatory insufficiency, general physical decline and genetics. Reactive oxygen species play an important role for performing signaling functions in brain, and for auditory processing, therefore, the brain and the inner ear are susceptible to oxidative stress (Fujimoto and Yamasoba, 2014; Cobley et al., 2018).

Experimental studies have investigated the neurodegenerative process that has been observed in both the hippocampus and auditory system. Blast injury, which is a common etiology for sensorineural hearing loss and tinnitus (Dougherty et al., 2013), causes dysfunction of the microtubule-associated protein, tau. Du et al. (2016, 2017) have found that blast-induced tau accumulates in the rat hippocampus and neurons in both the peripheral and central auditory systems, spanning from the spiral ganglion to the auditory cortex (Du et al., 2016, 2017).

Because tauopathies and cochlear neurodegeneration share the common pathophysiological correlate of oxidative stress (Alavi Naini and Soussi-Yanicostas, 2015; Du et al., 2016, 2017; Tavanai and Mohammadkhani, 2017) have demonstrated that therapeutic intervention with antioxidants significantly decreased both pathologic tau accumulation and indications of ongoing neurodegeneration in the cochlea and auditory cortex of blast-exposed rats.

Park et al. (2018) have examined the long-term effects of noise-induced hearing loss on cognition in a 12-month follow-up study of C57BL/6 mice and observed hippocampal p-tau and lipofuscin (Park et al., 2018). Moderate hearing loss resulted in working and recognition memory impairments, along with hippocampal neuronal degeneration, which was indicated by increased p-tau and lipofuscin. Permanent impairments of recognition memory, which is a form of episodic memory that is capable of discriminating between a previously viewed object and a novel one, were observed in the hearing-impaired mice. They concluded that the data provided behavioral and histopathological evidence for a causal relationship between peripheral hearing loss and cognitive decline.

To assess the relevance among hearing ability, hippocampal volume, and cognitive functions, especially in memory domain, we supplementally conducted a subgroup analysis in 1070 subjects aged 60 and over who tested the Mini-Mental State Examination (MMSE). From the 30 items of the whole MMSE that address multiple cognitive domains, we extracted six items which are specifically related to memory function calculated by the following formula: (immediate recall of three words + delayed recall of three words). This subset score, named as a short form of MMSE (SMMSE), is considered to be efficient in screening dementia among older adults with a memory complaint (Haubois et al., 2011), and have reported being correlated with hippocampal volume (Dinomais et al., 2016). We also calculated subtracted scores (MMSE-SMMSE) to represent cognitive functions not related to memory. We conducted Pearson partial correlation analyses adjusting for the same confounding factors as the main analyses. The results demonstrated that all of the SMMSE, MMSE-SMMSE, and full MMSE scores are significantly correlated with the PTA speech, however, only the SMMSE scores showed a modest but significant correlation with the hippocampal volume (**Table 4**). Although this evaluation was simple and preliminary, the results suggest significant links among hearing ability, hippocampal volume, and memory function. One can argue that the significant correlation between the SMMSE score and the PTA speech might be simply due to hearing difficulty in understanding the question. However, we consider it is not probable because most of the participants (1060 of 1070) obtained a full score (3/3) for the immediate recall task indicating they could hear and understand the question. In addition, the results did not change if we analyzed using these 1060 subjects.

**Table 4:** A subgroup analysis to assess the relationship of cognitive function with hearing and with hippocampal volume, using Pearson partial correlation analyses (Only over 60 years old, *N* = 1070).

	Range	Cognitive domains assessed	PTA speech		Hippocampal volume	
				
			Partial correlation coefficient	*p*	Partial correlation coefficient	*p*
SMMSE score ^a)^	0–6	Memory	**-0.093**	**0.002**	**0.073**	**0.018**
(MMSE-SMMSE) score	0–24	Scores unrelated to memory	**-0.098**	**0.001**	0.010	0.742
MMSE score	0–30	Global cognition	**-0.120**	**<0.001**	0.041	0.181


*a) SMMSE score was calculated following the formula: (immediate recall of three words + delayed recall of three words). Adjusted for age; sex; smoking status; alcohol consumption; years of education; medical history of hypertension, hyperlipidemia, cardiac disease, and diabetes; and depressive symptoms. Bold values indicate statistically significant results.*

The present study had several limitations. First, the results seen in the present cross-sectional analyses are not sufficient for debating if there is a cause-and-effect relationship between hippocampal volume and peripheral hearing. The present analytical cross-sectional studies are useful for establishing preliminary evidence for a link between the two, but interpretation requires caution regarding the potential association. Further analyses by longitudinal design will be required in order to show temporal precedence of the cause (i.e., the cause must precede the effect). Second, information on pathological biomarkers, such as amyloid-beta (Aβ) and tau, which are critical in Alzheimer’s disease and other types of dementing disorders, was not available. However, atrophy of the medial temporal lobe observed in the hearing-impaired individuals might be different from that seen in Alzheimer’s disease, because we found no relationship between hearing and the entorhinal cortex, which is known to be a region that shows prominently progressive atrophy in the early stages of Alzheimer’s disease (Gómez-Isla et al., 1996; Du et al., 2004).Third, information on the APOE genotype, of which APOE4 is known as a risk factor of Alzheimer’s disease, was not analyzed. Forth, the relationships among hearing ability, hippocampal volume, and memory function still need to be elucidated because cognitive test scores, which directly assess memory function (e.g., logical memory in the Wechsler Memory Scale–Revised), were not available in this study.

Even considering the above limitations, the impact of hearing on hippocampal atrophy among Japanese community dwellers is noteworthy. The common link among hearing ability, hippocampal volume, and cognitive function and whether auditory corrections cause longitudinal changes on brain volume should be further investigated.

## Conclusion

In conclusion, we examined the relationship between hearing ability and regional brain tissue volume, specifically focusing on the volumes of the hippocampus, Heschl’s gyrus, and total GM, which was obtained by MRI in the samples of over 2000 community dwellers aged 40 and older. More degraded peripheral hearing that was assessed by pure-tone audiometry was significantly correlated with smaller hippocampal volume after adjusting for potential confounding factors, and the association was consistent through the auditory frequency ranges. The current results suggested that the presence of hearing loss after middle age could be a modifier of hippocampal atrophy.

## Author Contributions

YU, YN, and AN drafting the manuscript, and taking responsibility for the accuracy of the data analysis. YU, YN, SS, CT, RO, FA, and HSh contribution to collecting data and statistical analyses. YN, TK, KI, and AN taking responsibility for brain MRI volume measurements and analyses. HSh and FA contribution to study concept and design of NILS-LSA. HSu and MS advice for interpretation of results. YU, SS, RO, and HSh: obtaining funding. All the authors contributed to the interpretation of the data and approved the final version of the manuscript before submission and will approve the final version to be published.

## Conflict of Interest Statement

The authors declare that the research was conducted in the absence of any commercial or financial relationships that could be construed as a potential conflict of interest.

## Footnotes

1 http://surfer.nmr.mgh.harvard.edu

## Abbreviations

GM: gray matter
MRI: magnetic resonance imaging
NILS-LSA: National Institute for Longevity Sciences-Longitudinal Study of Aging
PAF: population attributable fraction
PTA: pure-tone average
WM: white matter
